# Results of the preclinical multicenter randomized controlled paclitaxel-induced neuropathy prevention replication study (PINPRICS)

**DOI:** 10.1186/s13104-025-07206-2

**Published:** 2025-04-08

**Authors:** Wolfgang Boehmerle, Tim Hagenacker, Markus Leo, Linda-Isabell Schmitt, Helmar C. Lehmann, Ines Klein, Regina Stegherr, Frank Konietschke, Matthias Endres, Petra Huehnchen

**Affiliations:** 1 Klinik und Hochschulambulanz für Neurologie, Charité - Universitätsmedizin Berlin, corporate member of Freie Universität Berlin and Humboldt-Universität zu Berlin, Charitéplatz 1, 10117 Berlin, Germany; 2 Berlin Institute of Health at Charité, Universitätsmedizin Berlin, Anna-Louisa-Karsch Straße 2, 10178 Berlin, Germany; 3 Charité - Universitätsmedizin Berlin, corporate member of Freie Universität Berlin, Humboldt-Universität zu Berlin, NeuroCure Cluster of Excellence, Charitéplatz 1, 10117 Berlin, Germany; 4 Department of Neurology and Center for Translational Neuro- and Behavioral Science, University Hospital Essen, Hufelandstr. 55, 45147 Essen, Germany; 5 Department of Neurology, Medical Faculty, University Hospital Cologne, 50937 Köln, Germany; 6 Institut für Biometrie und Klinische Epidemiologie, Charité – Universitätsmedizin Berlin, corporate member of Freie Universität Berlin, Humboldt-Universität zu Berlin, 10117 Berlin, Germany; 7 Center for Stroke Research Berlin, Charité - Universitätsmedizin Berlin, corporate member of Freie Universität Berlin, Humboldt-Universität zu Berlin, Charitéplatz 1, 10117 Berlin, Germany; 8 German Center for Neurodegenerative Diseases (DZNE), Partner site, Berlin, Germany; 9 Partner site, German Center for Cardiovascular Research (DZHK), Berlin, Germany; 10 Partner site, German Center for Mental Health (DZPG), Berlin, Germany

**Keywords:** Chemotherapy-induced polyneuropathy, Neuropathic pain, Neuroprotection, Preclinical replication study

## Abstract

**Objective:**

Chemotherapy-induced peripheral neuropathy (CIPN) is a frequent and serious side effect of many cytotoxic drugs, including paclitaxel. Despite the identification of treatment options in animal models, clinical trials for the treatment or prevention of CIPN have been negative. Major challenges for successful clinical translation of preclinical data include a lack of reproducibility and randomization, small sample sizes and insufficient statistical tests. We therefore conducted a confirmatory, preclinical multicenter randomized controlled replication trial to test the safety and efficacy of three drugs for preventing paclitaxel-induced polyneuropathy: (1) nilotinib, (2) lithium carbonate and (3) interleukin-6-neutralizing antibodies. We preregistered the data analysis plan as well as the two-step study protocol: the optimal doses of the three compounds were assessed first and then tested in a mouse breast cancer xenograft model to compare safety and efficacy.

**Results:**

Unfortunately, toxicity of intraperitoneally administered nilotinib in combination with paclitaxel was observed, and higher-than-expected tumor growth resulted in a lack of power when the trial was analyzed. Thus, although lithium carbonate and IL-6-neutralizing antibodies tended toward neuroprotection, the differences between these groups were not statistically significant. However, the PINPRICS study ultimately still provides important lessons with regard to the planning and conduction of multicenter preclinical trials.

**Supplementary Information:**

The online version contains supplementary material available at 10.1186/s13104-025-07206-2.

## Introduction

**Fig. 1:**
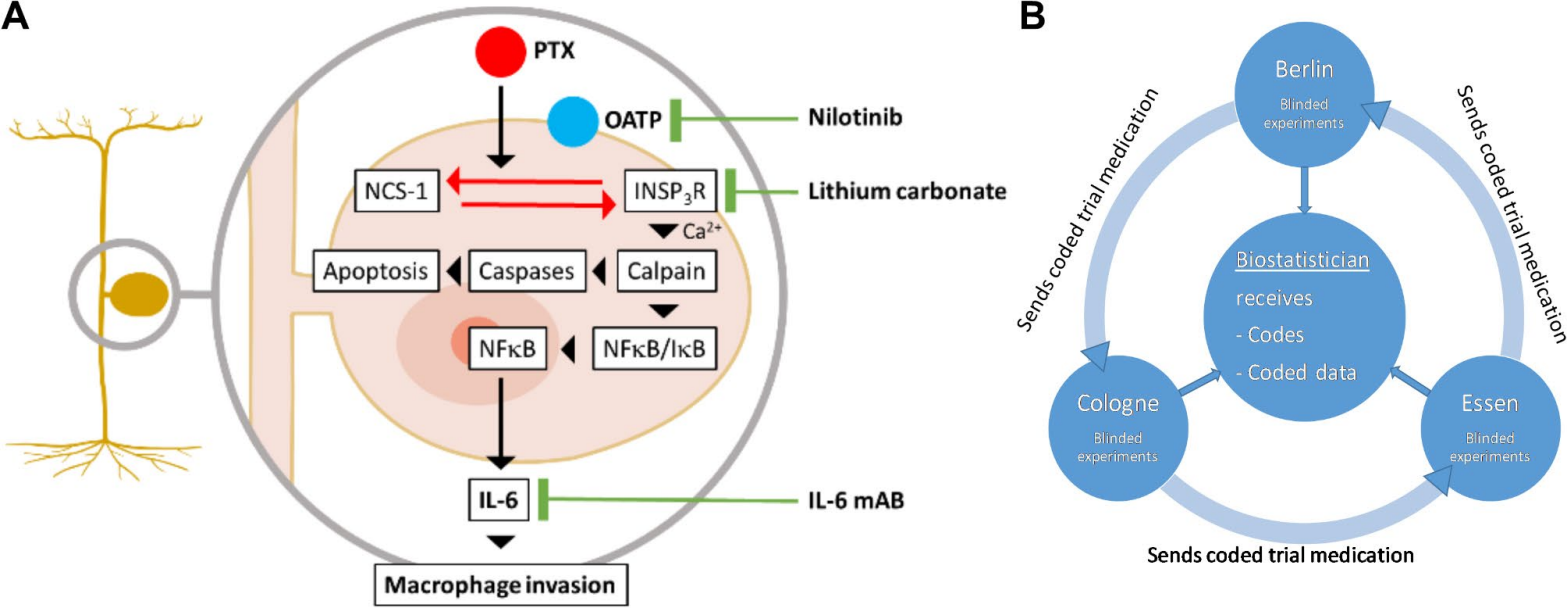
Overview of the molecular targets and organizational structure of the PINPRICS trial. **A**) Summary of seminal molecular mechanisms involved in the pathogenesis of paclitaxel-induced polyneuropathy and possibilities for pharmacological modulation with repurposed drugs. Modified from [10]. **B**) Interaction between the trial centers in the multicentric preclinical replication study PINPRICS: Study centers exchange coded trial medication and report codes and blinded measurements only to the biostatistician. All the individuals involved in the experiments and data analysis were fully blinded. Pseudonomyized data are entered into a central RedCap database via a web interface. Abbreviations: Ca^2+^, calcium; IkB, inhibitor of kappa B; IL-6, interleukin 6; IL-6 mAB, monoclonal antibody against interleukin 6; InsP_3_R, inositol 1,4,5, trisphosphate receptor; NCS-1, neuronal calcium sensor 1 protein; NFkB, nuclear factor kappa B; OATP, organic-anion-transporting polypeptide; PTX, paclitaxel

Chemotherapy-induced peripheral neuropathy (CIPN) is among the most common side effects of cytotoxic chemotherapy and presents an immense yet unmet medical need. Paclitaxel (PTX) is a cytotoxic drug that is frequently used to treat solid tumors but causes CIPN in 59–93% of treated patients (reviewed in [1]). In terms of prevention and treatment trials for CIPN, the results of a positive phase III study of duloxetine provided evidence for symptomatic pain relief [2], but prevention and causal treatment trials for CIPN were neutral (summarized in [3]). Treatment options for CIPN are therefore presently limited to symptomatic and supportive treatments. Preclinical research has elucidated a number of pathophysiological mechanisms that mediate PTX-induced damage to dorsal root ganglia neurons of the sensory nervous system. Among published preclinical neuroprotective strategies, three involve drugs with market authorization for other indications, which facilitates clinical translation (summarized in Fig. 1A). These drugs target the following: (1) *The entrance* of PTX into sensory neurons via organic-anion-transporting polypeptides (OATP) can be targeted with nilotinib [4]. (2) *Intracellular* induction of calcium dyshomeostasis [5–9], which can be prevented with lithium ions, and (3) a *secondary neuroimmune interaction* mediated by the cytokine IL-6 that can be addressed with IL-6 neutralizing antibodies [10]. To test and compare the safety and efficacy of these interventions, we designed a multicentric preclinical replication trial in mice, which consisted of an initial dose confirmation study for each candidate and a subsequent prevention trial in a mouse breast cancer tumor transplant model.

## Materials and methods

A detailed description of the materials and methods is provided in the supplemental materials and methods section.

### Trial design of the PINPRICS trial

PINPRICS is a confirmatory preclinical multicenter randomized controlled trial. The aim of the PINPRICS trial was to replicate previously observed neuroprotective effects of substances with a market authorization for another indication (“repurposing”). To minimize bias, experiments were performed with a third of the planned animals in three centers with documented experience in animal models of paclitaxel-induced polyneuropathy (Berlin, Essen and Cologne, all in Germany). To ensure proper blinding, we decided to organize the consortium in a “hub and spoke” configuration with an independent biostatistician serving as the hub who received group allocations and datasets from blinded investigators (Fig. 1B). The PINPRICS trial consisted of an initial dose-finding phase (PINPRICS-DC) followed by the PINPRICS prevention study (PINPRICS-PS) in a breast cancer mouse xenograft model. The primary efficacy endpoint was defined as changes in the sensory nerve action potential amplitude of the tail nerve (see below “electrophysiology”). The trial was preregistered at www.animalstudyregistry.org and the open science framework initiative (www.osf.io). The full text is available at 10.17590/asr.0000202 and 10.17605/OSF.IO/A2KBQ.

### Ethics statement

This study conformed to governmental and institutional animal welfare guidelines and was approved by the official animal ethics committee of Berlin and North Rhine-Westphalia (Landesamt fuer Gesundheit und Soziales Berlin, Germany; Landesamt für Natur, Umwelt und Verbraucherschutz Nordrhein-Westfalen (LANUV), Germany) prior to the execution of the experiments. The protocol was optimized in accordance with the 3R principles, and every effort was made to minimize suffering.

### Animals

A total of 198 twelve- to sixteen-week-old female BALB/C mice, all purchased from Charles River (Sulzfeld, Germany), were used for this study. To reduce possible litter effects, the animals were reassigned to different cages upon arrival at the animal housing facilities with the help of randomly generated numbers. The mice were housed in groups of five and allowed food and water *ad libitum*. The animals were maintained on a 12:12 h light/dark cycle (7 am − 7 pm). Behavioral testing (described below) was conducted between 10 am and 6 pm. If an injection was administered on the same day as the behavior tests, it was administered only after all testing had been completed. If the injections and electrophysiological measurements coincided (vide infra), injections were given while the animals were anesthetized. The general well-being of the mice was assessed daily, and their weights were recorded regularly. Animals were killed by decapitation under deep isoflurane anesthesia at the time points described in detail in the results section or in the event of excessive tumor growth, distress, pain or suffering according to predefined humane endpoints before the end of the experiment (premature killing).

### Drug injection protocol

*Paclitaxel* (Biomol GmbH, Germany) was administered as described previously [6, 11]. In short, a stock solution of paclitaxel was prepared at each study center in Kolliphor EL: ethanol (1:1, Merck, Darmstadt, Germany) at a concentration of 6 mg/ml. The stock solution was then assigned a numerical code for the purpose of blinding and sent on blue ice to the other study centers (Fig. 1B). Each study center then prepared the final injection solution on the days of injection by diluting it 1:3 with 0.9% NaCl to a maximum injection volume of 10 ml/kg body weight, yielding a final paclitaxel dose of 20 mg/kg body weight.

*Kolliphor EL: ethanol* (1:1) (Merck, Darmstadt, Germany) was used as a solvent for paclitaxel and in the vehicle control group.

*Nilotinib* (Fisher Scientific GmbH, Germany) was injected intraperitoneally at doses of up to 500 mg/kg BW approximately 30 min before each paclitaxel injection. A stock solution of nilotinib in DMSO at 50 mg/ml was prepared for this purpose. This mixture was further diluted with 0.9% NaCl, and the final solutions were coded and sent as ready-to-use solutions to the other study centers.

*Lithium carbonate* (Carl Roth GmbH, Germany) was administered intraperitoneally at doses ranging from 2.6 to 64 mg/kg BW approximately 30 min before each paclitaxel injection. Lithium carbonate was dissolved at concentrations of 6.4 mg/ml (corresponding to 64 mg/kg body weight lithium carbonate dose), 1.28 mg/ml (corresponding to 12.8 mg/kg body weight lithium carbonate dose) or 0.26 mg/ml (corresponding to 2.6 mg/kg body weight lithium carbonate dose) in 0.9% NaCl. All the solutions were coded and sent as ready-to-use solutions to the other study centers, and an injection volume of 10 ml/kg body weight was applied for all doses.

The *IL-6 neutralizing antibody MAB406* (R&D Systems, Minneapolis, MN) was evaluated in PINPRICS-DC at doses ranging from 1 to 25 mg/kg BW. MAB406 was dissolved at 0.1 mg/ml (corresponding to 1 mg/kg body weight dose), 0.5 mg/ml (corresponding to 5 mg/kg body weight dose) or 2.5 mg/ml (corresponding to 25 mg/kg body weight dose) in 0.9% NaCl, coded and sent to the other study center as a ready-to-use solution. The application was carried out with an injection volume of 10 ml/kg body weight intraperitoneally once a week. To achieve complete blinding, 10 ml/kg body weight 0.9% NaCl was administered on the other eight injection days.

### Tumor xenograft model

For the PINPRICS-PS study, BALB-C mice with a breast cancer xenograft, were used as described previously [12]. In short, 4T1 cells (ATCC, Manassas, VA) have low immunogenicity but are highly malignant human breast cancer cells. The cells were injected at a dose of 5 × 10^4^ cells into the subcutaneous fatty tissue of the lateral mammary glands of female BALB/c mice. The primary tumor was regularly measured with a caliper, and treatment with chemotherapy and neuroprotective substances started seven days after tumor cell injection. To ensure that the primary endpoint on day 42 could be measured in the Kolliphor EL: ethanol group, only 2.5 × 10^4^ 4T1 cells were injected into the animals in this group. The blinding of the groups was maintained by having these injections carried out by persons not otherwise involved in the experiment.

### Cell culture experiments

#### Culture of 4T1 cells

4T1 cells were obtained from American Type Culture Collection (ATCC, Manassas, VA) and cultivated as recommended by the manufacturer in RPMI-1640 medium (Merck/Sigma‒Aldrich, Germany) supplemented with 10% fetal bovine serum and 1% penicillin/streptomycin (both from BiochChrom, Germany). The cultures were maintained at 37 °C in a humidified atmosphere with 5% CO_2_. Cultured 4T1 cells were used in the tumor xenograft model and were assessed for sensitivity to paclitaxel treatment.

#### Cell viability assays

The cytotoxicity of paclitaxel in cultured 4T1 cells was assessed as described previously [13].

### Behavior analysis

Prior to the experiment, the animals were familiarized with the investigator by handling them for five days according to a previously specified handling protocol prior to the start of the experiment. During the experiments, the experimenters randomly selected cages and animals in a laboratory with soundproof chambers. The investigators adhered to standard operating procedures that built upon existing lab routines and which were consented in a series of video conferences.

#### Rotarod

In the PINPRICS-PS subtrial, we assessed motor coordination via the rotarod performance test [6, 11]: Mice were placed on a rotating rod in individual compartments, with walls on both sides and in front of them (TSE Systems GmbH, Germany). Within 300 s, the speed of the rotating rod increased from four rounds per minute (rpm) to a maximum speed of 40 rpm, and the latency for the animal to fall off the rod was automatically recorded by a floor sensor. To allow the mice to learn the task, the animals were trained for four days with three trials per day, with a daily increase in the maximum time spent on the rod from 70 s per trial on day one to 140 s per trial on day 2, 210 s per trial on day 3 and finally 300 s per trial on day four. The mice that fell off the rod during training within the designated time were gently placed back on the rod. The mice were brought back to their home cage from the moving rod only to prevent the animals from exhibiting dropping behavior.

#### Von Frey hair test

In the PINPRICS-PS subtrial, mechanical allodynia was assessed via von Frey hairs and the up and down method, as described previously [14] to determine the 50% probability withdrawal threshold. The mice were placed under an inverted plastic cage with a wire-mesh floor. Investigators underwent extensive training to apply the filaments to the center of the hind paws, gradually increasing pressure. Poking either hind paw evoked a flexion reflex followed by a clear withdrawal response. The value of each filament that evoked a withdrawal response was noted, and the next lower value was used for the next round of testing.

### Nerve conduction studies

In PINPRICS-DC and PINPRICS-PS subtrials, the quantification of nerve damage measured by changes in tail nerve sensory nerve action potential (SNAP) amplitudes on day 42 served as the primary efficacy endpoint. Additional measurements were performed at baseline and at days 14 and 28. The nerve conduction velocity (NCV) and SNAP of the caudal nerve were recorded under isoflurane anesthesia (1.3-1.7% in 50% O_2_) with electromyography and nerve conduction systems (Berlin: Neurosoft 3102evo, Schreiber & Tholen Medizintechnik, Germany; Cologne and Essen: Dantec Keypoint G3, Natus, Planegg, Germany).

### Sample size calculation and statistical analysis plan

#### Sample size calculation

We performed a sample size calculation for the PINPRICS-DS and PINPRICS-PS subtrials, which are described in detail in the supplemental materials and methods. In short, sample size calculations were performed with R and the mvtnorm library [15], yielding a sample size of *n* = 9 per group for the PINPRICS-DC experiment and *n* = 15 per group for the PINPRICS-PS experiment (5 groups, alpha error: 0.05, power 0.91).

#### Electronic trial database

Study data were collected and managed via REDCap electronic data capture tools hosted at a server of Charité Universitätsmedizin Berlin [16, 17].

#### Statistical analysis and data presentation

The data were received by an independent statistician (Fig. 1B) and pooled for analysis. SNAP, rotarod and von Frey values were normalized to the center baseline prior to pooling the data. The data are expressed as the means ± standard deviations respectively medians with ranges, and the manuscript was written in accordance with ARRIVE guidelines [18]. Statistical analysis of the differences between the treated and control groups was performed as prespecified with a multiple contrast test via a linear regression model adjusted for the baseline SNAP amplitude and center by means of a covariate (ANCOVA) with the multcomp package in R. *p* < 0.05 was considered statistically significant.

## Results

### PINPRICS dose confirmation

**Fig. 2:**
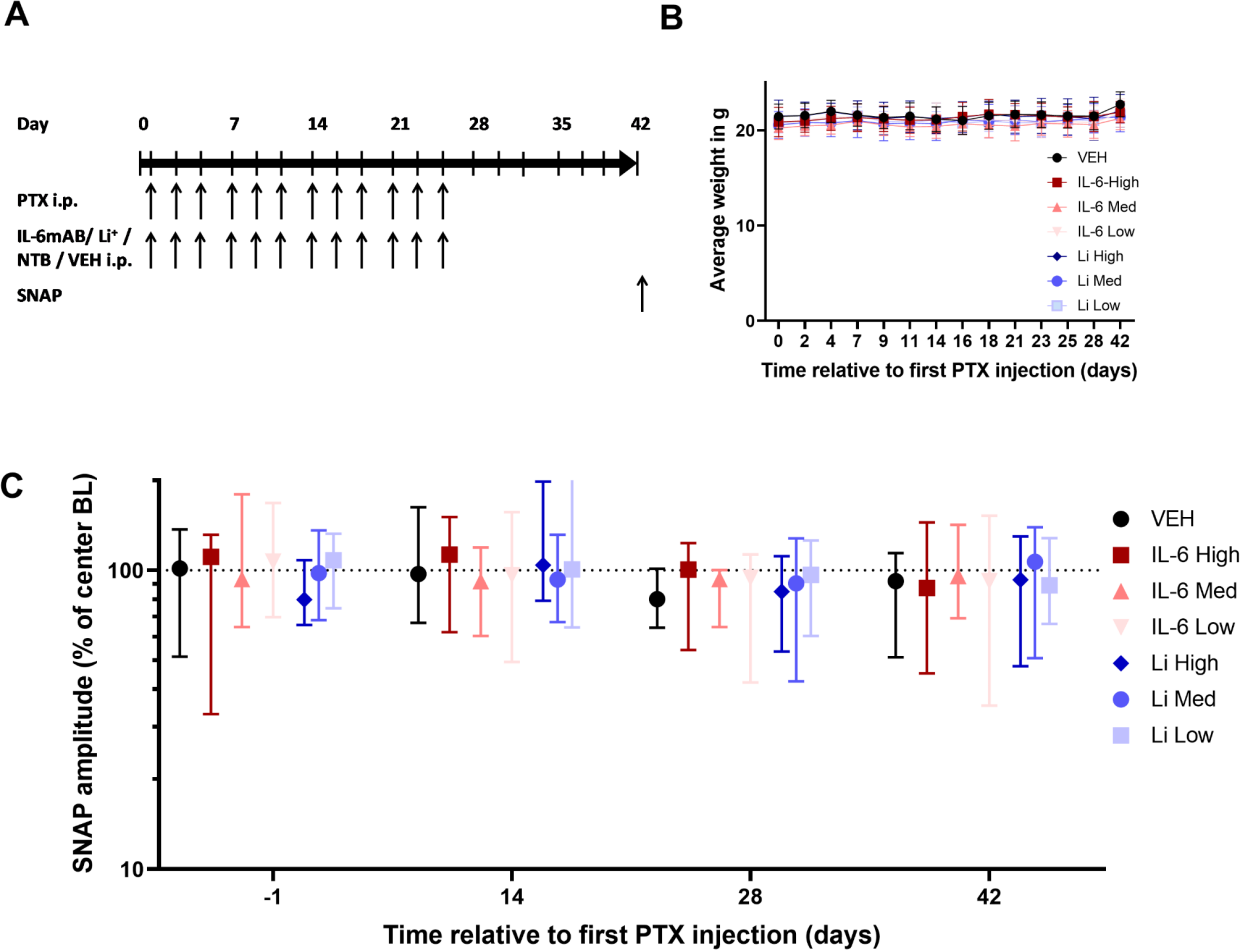
Results from the PINPRICS-DC subtrial. (**A**) Schematic representation of the trial design for the PINPRICS-DC dose confirmation study. The substances were all applied intraperitoneally three times a week (Monday-Wednesday-Friday) for four weeks. On days − 1, 14, 28 and 42 of the experiment, the sensory nerve action potential in the caudal nerve was measured. The animals were killed on day 42 by decapitation under deep isoflurane anesthesia. (**B**) The average weight was comparable among all the experimental groups, and none of the animals experienced weight loss exceeding 20% of the baseline weight. (**C**) Analysis of SNAP amplitudes normalized to center baselines. Animals treated with paclitaxel and vehicle presented the greatest decrease in SNAP amplitudes; however, the treatment effects were obscured by variance exceeding the initial assumptions. Initial sample size in B + C; *n* = 9 per group (VEH *n* = 8 from day 21). Abbreviations: BL, baseline; IL-6mAB/IL-6, monoclonal IL-6 antibody MAB406; i.p., intraperitoneal; Li^(+)^, lithium carbonate; NTB, nilotinib; PTX, paclitaxel; SNAP, sensory nerve action potential; VEH: Vehicle

The treatment schedule of the dose-confirmation study (PINPRICS-DC) is summarized in Fig. 2A. Overall, 3 doses per potentially neuroprotective drug (lithium carbonate, Il-6 neutralizing antibodies and nilotinib) were compared to the vehicle control group, and all the animals were treated with paclitaxel. In the first trial center, three groups of test animals were severely impaired after the first round of injections and had to be sacrificed. Owing to this unexpected development, selective unblinding was performed, and all of the prematurely killed animals belonged to the three nilotinib groups. We therefore decided to reduce and optimize the nilotinib doses in subsequent experiments at the remaining two trial centers and performed the experiments with nilotinib doses of 0.4, 2 and 10 mg/kg BW. Nevertheless, animals from three test groups had to be removed from the trial prematurely again. Unblinding after completion of all the experiments confirmed that, again, only nilotinib-treated animals were affected, which suggests that the combination therapy of paclitaxel with intraperitoneal nilotinib causes supralinear toxicity, i.e., a disproportionate increase in toxic effects relative to the dose of the two drugs. Animals from the other experimental groups presented no clinical signs of toxicity and had comparable body weights (Fig. 2B). Normalized data revealed an almost doubled standard deviation of SNAP compared with single center datasets. We observed, for example, a vehicle baseline median of the normalized SNAP amplitude of 102% with a range [51 − 137%, *n* = 9] compared to a historic cohort examining the effects of IL-6 inhibition with the same technique of SNAP assessment [10], where a baseline median of 101% with a range [77 − 119%, *n* = 10] was observed (Fig. 2C). The median SNAP amplitude decreased in animals injected with paclitaxel and vehicle from 102% at baseline to 80% on day 28 (*n* = 8) but then recovered to 92% of the baseline value by day 42 (*n* = 8). Owing to the observed recovery, SNAP amplitudes on day 28, instead of day 42, were analyzed to determine optimal dosages for the subsequent PINPRICS-PS subtrial. At this time point, we observed a median SNAP of 85% [111% − 54%, *n* = 9] for the high-dose lithium carbonate group, 90% [128% − 43%, *n* = 9] for the medium-dose lithium carbonate group and 97% [126% − 60%, *n* = 9] for the low-dose lithium carbonate group on day 28. There was no statistically significant difference between the three doses, but the median SNAP amplitude was highest and the standard deviation was the smallest for the lowest dose of lithium carbonate, which is why we continued with the lowest dose of 2.6 mg/kg BW in the PINPRICS-PS subtrial. For the IL-6 neutralizing antibody, we observed a median SNAP of 101% [54 − 123%, *n* = 9] for the high-dose group, 94% [65 − 100%, *n* = 9] for the medium-dose group, and 94% [42 − 113%, *n* = 9] for the low-dose group. We continued as previously specified with the medium-dose group, as it had comparable efficacy to the high-dose group as well as the lowest standard deviation.

### PINPRICS prevention study

**Fig. 3:**
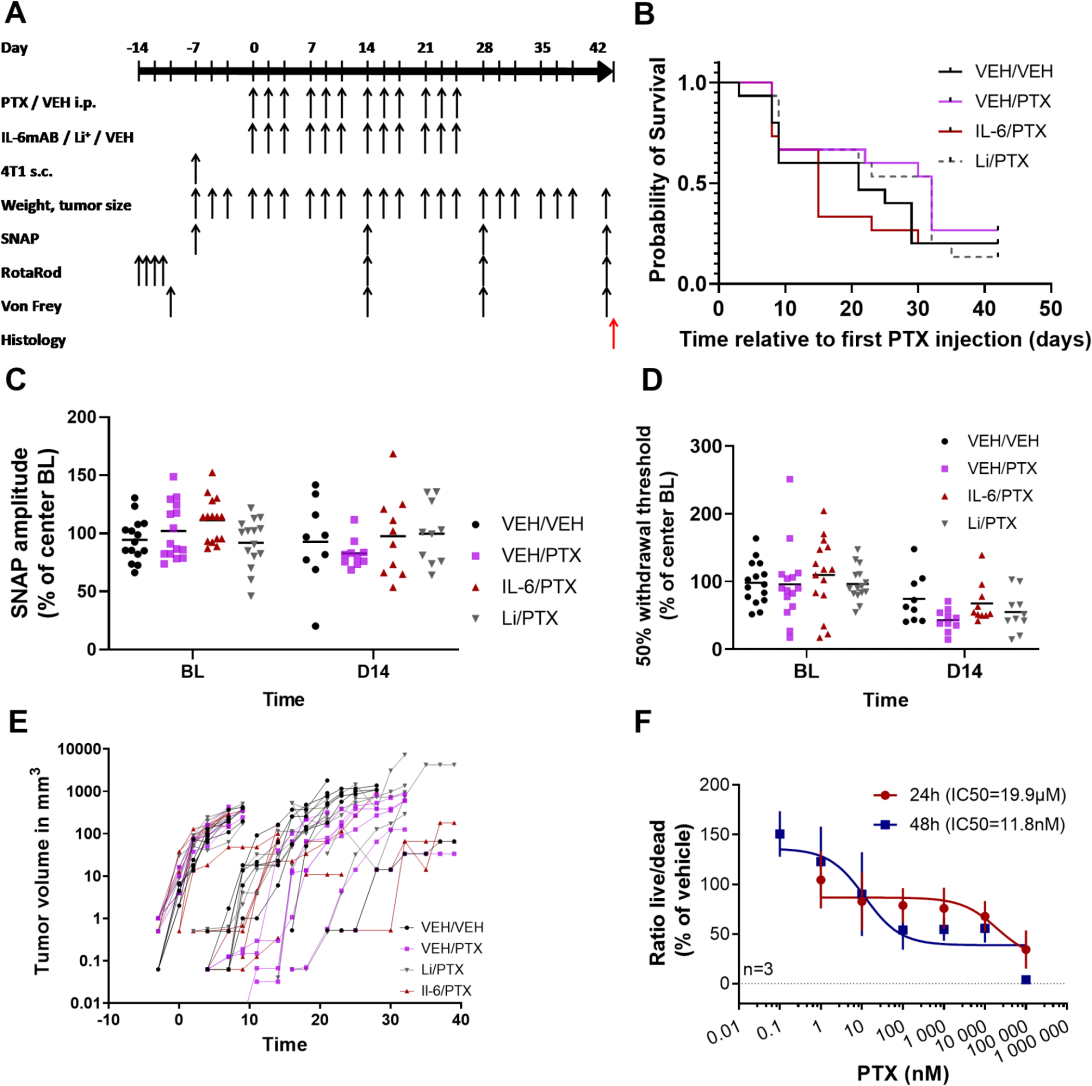
Results from the PINPRICS-PS subtrial. (**A**) Schematic representation of the trial design for the PINPRICS-PS study. One week prior to the start of paclitaxel therapy, the animals were transplanted with human 4T1 breast cancer cells by subcutaneous injection into the mammary fat pad (4T1 s.c.). Thereafter, weight and tumor size were closely monitored. The animals were sacrificed on day 42 by decapitation under deep isoflurane anesthesia. (**B**) Kaplan‒Meier plot of survival per treatment group. (**C**) Analysis of SNAP amplitudes of the caudal nerve normalized to center baselines at baseline (BL) and day 14 (VEH/VEH: *n* = 9; VEH/PTX *n* = 10; IL-6/PTX *n* = 10; Li/PTX *n* = 10). Owing to rapid tumor growth, there were not enough animals for analysis at later time points. The graph depicts the mean and individual values per group. (**D**) The 50% probability mechanical withdrawal threshold was measured with von Frey filaments and is presented at BL and day 14 normalized to the center baselines. Lower values indicate greater sensitivity to mechanical stimuli, which is indicative of mechanical allodynia. The graph depicts the mean and individual values per group (the sample size is identical to that in C). (**E**) Development of tumor volume over time. Each animal is represented with dots connected by a single line. Animals with tumors larger than 1500 mm^3^ were euthanized. The Y-axis uses a log10 scale. (**F**) Dose‒response curves of cultured 4T1 human breast cancer cells after 24 h and 48 h of incubation with increasing dosages of paclitaxel. The initial sample size was *n* = 15 per group. Abbreviations: 4T1 s.c, subcutaneous injection of 4T1 breast cancer cells; BL, baseline; Il6/IL-6mAB, monoclonal IL-6 antibody MAB406; i.p., intraperitoneal; Li^(+)^, lithium carbonate; PTX, paclitaxel; RR, RotaRod test; SNAP, sensory nerve action potential; VEH, vehicle; Von Frey, von Frey hair test

For the PINPRICS prevention study, BALB/c mice were transplanted with 4T1 breast cancer cells and subsequently treated with PTX (Fig. 3A). A tumor xenograft model was used to detect potential effects of neuroprotective drugs on the antineoplastic efficacy of PTX. The originally planned nilotinib group was omitted from the PINPRICS-PS subtrial because of the previously observed toxicity. At all the trial sites, tumor growth in the experimental animals was more rapid than previously published (Fig. 3E), and 49 animals had to be sacrificed prematurely according to predefined humane endpoints. Overall survival, as analyzed with the Kaplan‒Meier estimator, was highest in paclitaxel treated animals with an add on medication of lithium carbonate or vehicle (Fig. 3B). On day 14, SNAP amplitudes in the vehicle/paclitaxel group were lower (VEH/PTX, 83% of baseline ± 12%, *n* = 10) than those in the vehicle/vehicle group (VEH/VEH, 93% of baseline ± 37%, *n* = 9), the lithium carbonate/paclitaxel group (Li/PTX, 100% of baseline ± 26%, *n* = 10) and the IL6 neutralizing antibody/paclitaxel group (IL6/PTX, 98% of baseline ± 35%, *n* = 10). The differences between these groups were not statistically significant (Fig. 3C). We used the 50% probability withdrawal threshold with the von Frey method as an additional clinical endpoint. Animals treated with paclitaxel develop mechanical allodynia, i.e., increased sensitivity to mechanical stimuli [19]. When we analyzed von Frey values, as expected, we observed greater reductions in the 50% probability mechanical withdrawal threshold normalized to the center baseline in the VEH/PTX group (43% of baseline ± 16%, *n* = 10) than in the Li/PTX (55% of baseline ± 30%, *n* = 10), IL6/PTX (68% of baseline ± 30%, *n* = 10) and VEH/VEH control groups (74% of baseline ± 36%, *n* = 9; Fig. 3D), which again did not reach statistical significance. As expected from previous experiments with animal-models of CIPN [19] we observed comparable values for the motor function test rotarod between all groups (Supplemental Fig. 2).

To better understand the unexpected rapid tumor growth (Fig. 3E), the sensitivity of the 4T1 cells used in our study to paclitaxel in vitro was characterized. Dose response experiments revealed a calculated IC_50_ value of 19.9 µM after a 24-hour incubation period and 11.8 nM after 48 h of incubation with paclitaxel (Fig. 3F).

## Discussion

This preclinical confirmatory randomized controlled trial in mice with three drugs and three sites yielded the following major results: First, we observed a previously unreported supralinear toxicity of intraperitoneally applied nilotinib together with paclitaxel. Second, we observed an approximately twofold increase in the standard deviation of the SNAP measurements compared with a previous single-center assessment. Third, the prespecified analysis of PINPRICS-DC and PINPRICS-PS, although showing a trend toward neuroprotection for lithium carbonate and IL-6 neutralizing antibodies, yielded no significant results.

Even though the primary study objective in terms of a validated neuroprotective comedication could not be achieved, the lessons learned from the PINPRICS study hold great potential to inform the design and execution of future preclinical multicenter randomized clinical trials. Although rigorous advanced statistical planning of the experiments has been performed as recommended by previous preclinical RCTs [20], the greatest error in hindsight was the assumption that the variance of SNAP amplitude measurements, which served as the primary endpoint in both parts of the PINPRICS study, would be comparable to data obtained from a single site. An initial experimental protocol with much greater variance was rejected by institutional animal welfare experts because it was deemed too speculative. On the basis of the data from the PINPRICS experiments, it appears safe to calculate 2–3 times the variance observed in a single center in the future. The second most relevant complication arose from an experimental design that was very similar but not identical to experiments previously performed in participating laboratories. The reason for this approach was that German animal welfare laws prohibit duplicate or repeat attempts at animal experiments. In the context of the PINPRICS trial, an exact replication would have been able to avoid the observed nilotinib toxicity as well as accelerated tumor growth, which eventually prevented us from successfully concluding the prespecified analysis.

Despite the challenges outlined above, the established organizational structure around otherwise not involved statistical experts, the central study data collection tool based on the REDCap platform as well as the blinding strategy all worked largely in favor of the trial and should be considered in future multicenter replication trials.

### Limitations

First, the experimental phase of the PINPRICS trial started shortly after the onset of the SARS-CoV-2 pandemic, which meant that the trial could not proceed as planned. Travel restrictions led to a change in the planned meetings, preventing in-person meetings and workshops from being held on site. The extent to which the lack of hands-on training could have affected the variance of the test results remains speculative, but the assumption that it did appears plausible.

Second, a neuroprotective effect in paclitaxel-treated mice receiving 100 mg/kg BW nilotinib orally was demonstrated previously [4]. Owing to the necessity of applying the IL-6 antibody intraperitoneally, it was essential for blinding to use an identical route of administration for all the tested drugs. Given the bioavailability of approximately 50% nilotinib in mice [21], an intraperitoneal dose of 50–100 mg/kg body weight should have been safe and effective and was indeed used in different disease models with doses of up to 50 mg/kg BW [22, 23]. In the PINPRICS-DC trial, much lower doses were not tolerated, suggesting supralinear toxicity when nilotinib was coadministered with paclitaxel. A similar observation was made in acetaminophen-treated mice [24].

Third, in the PINPRICS-PS trial tumor growth, which was much faster than published results for less aggressive chemotherapy regimens [25, 26], was observed. Owing to the remaining low number of animals, no statistical significance could be detected according to the prespecified statistical analysis plan. Exploratory data analysis of the early time point revealed a trend that supported the hypothesis but again yielded no significant findings due to low sample sizes.

## Electronic supplementary material

Below is the link to the electronic supplementary material.

Supplementary Material 1

Supplementary Material 2

## Data Availability

The datasets supporting the conclusions of this article are included within the article and in the supplemental files.

## Abbreviations

BW: Body weight
CIPN: Chemotherapy-induced peripheral polyneuropathy
NCV: Nerve conduction velocity
PTX: Paclitaxel
SNAP: Sensory nerve action potential amplitudest
