# Supplementary material for: Results of the preclinical multicenter randomized controlled paclitaxel-induced neuropathy prevention replication study (PINPRICS)
Source: BMC Res Notes. 2025 Apr 8;18:145. doi: 10.1186/s13104-025-07206-2 (PMC11978143; doi:10.1186/s13104-025-07206-2)
Supplement: Supplementary file 2 — Supplementary Material 2 [file 13104_2025_7206_MOESM2_ESM.docx]

**Supplemental information**

**Supplemental Figure 1:**


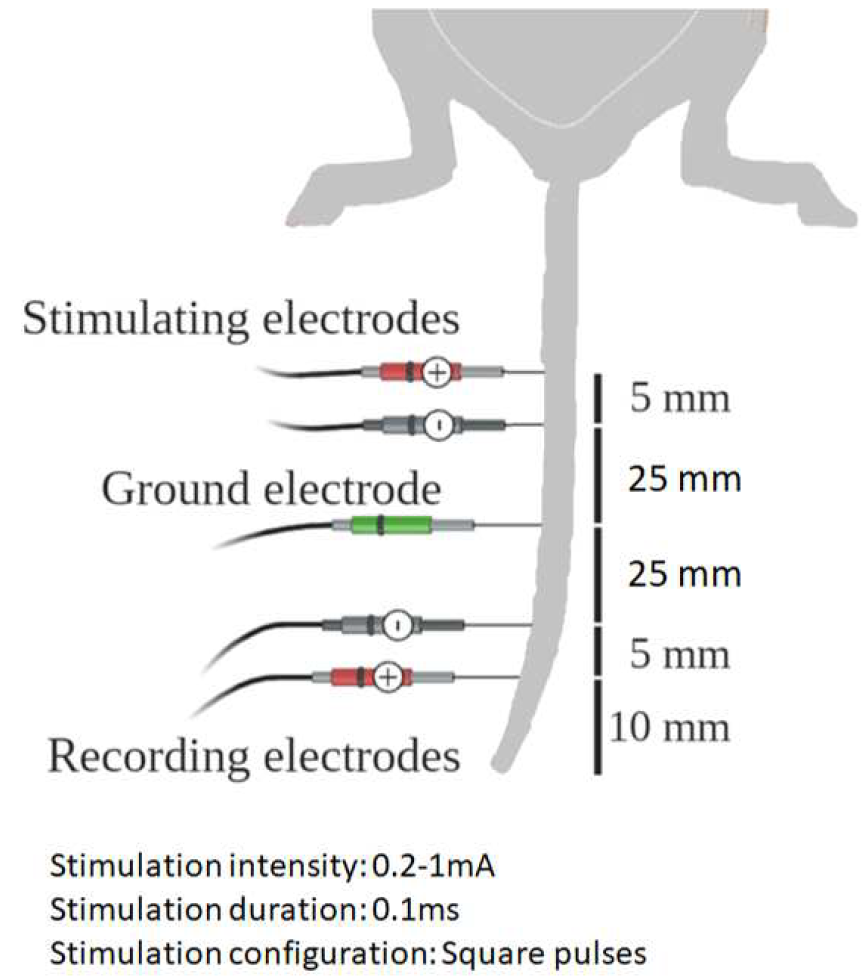


*Supplemental Figure 1: Position of the stimulation and recording electrodes as well as parameters used for the sensory nerve action potential (SNAP) and sensory nerve conduction velocity(SCV) recordings in mouse tail nerves*.

**Supplemental Figure 2:**

*Supplemental Figure 2: Results from the rotarod test. Time on the rotating rod was measured and is presented at BL and day 14 normalized to the center baselines. The graph depicts the mean and individual values per group (Sample size: VEH/VEH: n=9; VEH/PTX n=10; IL-6/PTX n= 10; Li/PTX n=10). Abbreviations: BL, baseline; IL-6: monoclonal IL-6 antibody MAB406; Li^(+)^: lithium carbonate; PTX: paclitaxel; VEH: vehicle.*

**Supplemental Materials and Methods:**

*Trial design of the PINPRICS trial*

The multicenter preclinical replication trial (PINPRICS) was pre-registered at [www.animalstudyregistry.org](http://www.animalstudyregistry.org) and the open science framework initiative (www.osf.io). Full text is available under DOI:10.17590/asr.0000202 and DOI:10.17605/OSF.IO/A2KBQ. PINPRICS is a confirmatory preclinical multicenter randomized control trial. The aim of the PINPRICS trial was to replicate previously observed neuroprotective effects of substances with a market authorization for another indication (“re-purposing"). In order to minimize bias, experiments were performed in three centers with documented experience in animal models of paclitaxel-induced polyneuropathy (Berlin, Essen and Cologne, all in Germany). To ensure proper blinding we decided to organize the consortium in a “hub and spoke” configuration with an independent biostatistician serving as hub and group allocations and datasets were received from blinded investigators (Figure 1B). In order to streamline the interaction and ensure proper data management including audit trails, all experimental data was entered in a REDCap database. The PINPRICS protocol involved two phases, an initial phase to identify the optimal dose (PINPRICS-DC) followed by the actual neuroprotection trial in a tumor xenograft model (PINPRICS-PS). The sample size calculation is presented below and each site performed experiments on 33% of animals. We chose an injection protocol with 12x 20mg/kg body weight paclitaxel as previously established that mimics the human “paclitaxel weekly” treatment of breast cancer patients [[9](#_ENREF_9)]. Due to the published treatment effects in animal models [[4](#_ENREF_4)], the calculated sample size was **n=3** per center and group for PINPRICS-DC and **n=5** per center and group for PINPRICS-PS. Because in-person meetings were not possible due to the SARS-COV2 pandemic, several video conferences were performed with all sites (Berlin, Essen, Cologne) to standardize relevant study details, harmonize experimental conditions, and establish standard operating procedures. Originally, we had planned to also organize a “hands on” training workshop with all experimenters, which however had to be cancelled due travel restriction during the pandemic.

**Animals**

A total of 198 twelve to sixteen week-old female BALB/C mice, all purchased from Charles River (Sulzfeld, Germany), were used for this study. In order to reduce possible litter effects, animals were reassigned to different cages upon arrival in the animal housing facilities with the help of randomly generated numbers. Mice were housed in groups of five and allowed food and water *ad libitum*. The animals were maintained on a 12:12 hour light/dark cycle (7 am – 7 pm). Behavioral testing was conducted between 10 am and 6 pm. If an injection was administered on the same day as behavior tests, it was administered only after all testing had been completed. The general well-being of the mice was assessed daily and weight was recorded regularly.

**Drug Injection protocol**

Paclitaxel (Biomol GmbH, Germany) was administered by 12 intraperitoneal injections of 20 mg/kg body weight three times a week (Monday, Wednesday, Friday) for a total of 4 weeks [[4](#_ENREF_4), [9](#_ENREF_9)]. A stock solution of paclitaxel was prepared at each study center in Kolliphor EL:ethanol (1:1) at a concentration of 6 mg/ml. The stock solution was then assigned a numerical code for the purpose of blinding and sent on blue ice to the respective other study center (Figure 1B). Each study center then prepared the final injection solution on the days of injection by diluting 1:3 with 0.9% NaCl to a maximum injection volume of 10 ml/kg body weight. The model we used with an injection of 20 mg/kg body weight paclitaxel is comparable to cytotoxic treatment in humans for breast cancer (corresponds to a human equivalent dose of 12x 65 mg/m2 body surface area).

Kolliphor EL : ethanol (1:1) (Sigma-Aldrich, Germany) was used as a solvent for paclitaxel and thus as a substance in a vehicle control group in the prevention study. Kolliphor EL:ethanol was sent along the paclitaxel stock solution in coded form to the respective other study center. There the stock solution was diluted 1:3 with 0.9% NaCl to the final concentration and an injection volume of max. 10 ml/kg body weight was injected intraperitoneally.

Nilotinib (Fisher Scientific GmbH, Germany) is a tyrosine kinase inhibitor which among others is used in the treatment of chronic myeloid leukemia [[10](#_ENREF_10)]. Nilotinib was injected intraperitoneally in doses of 20 mg/kg body weight, 100 mg/kg body weight, 500 mg/kg body weight, respectively after detecting unexpected toxicity 0.4 mg/kg body weight, 2 mg/kg body weight and 10 mg/kg body weight doses approx.. 30 min before each paclitaxel injection to determine the optimal dose in the PINPRICS-DC trial. A stock solution of nilotinib in DMSO at 50 mg/ml was prepared for this purpose. This was further diluted with 0.9% NaCl and the final solutions were coded and sent as ready-to-use solutions to the respective other study center.

Lithium carbonate (Carl Roth GmbH, Germany) was administered intraperitoneally at 2.6 mg/kg body weight, 12.8 mg/kg body weight or 64 mg/kg body weight approx. 30 min before each paclitaxel injection. For this purpose, lithium carbonate was dissolved at 6.4 mg/ml (corresponds to 64 mg/kg body weight lithium carbonate), 1.28 mg/ml (corresponds to 12.8 mg/kg body weight lithium carbonate) or 0.26 mg/ml (corresponds to 2.6 mg/kg body weight lithium carbonate) in 0.9% NaCl. All solutions were coded and sent as ready-to-use solutions to the respective other study centers and applied there with an injection volume of 10 ml/kg body weight.

The IL-6 neutralizing antibody MAB406 (R&D Systems, Minneapolis, MN) recently proved effective in the prevention of paclitaxel-induced polyneuropathy [[8](#_ENREF_8)]. MAB406 was evaluated in PINPRICS-DC at doses of 1 mg/kg body weight, 5 mg/kg body weight and 25 mg/kg body weight with regard to its neuroprotective effect. For this purpose, MAB406 was dissolved at 0.1 mg/ml (corresponds to 1 mg/kg body weight), 0.5 mg/ml (corresponds to 5 mg/kg body weight) or 2.5 mg/ml (corresponds to 25 mg/kg body weight) in 0.9% NaCl, coded and sent to the respective other study center as a ready-to-use solution. The application was carried out with an injection volume of 10 ml/kg body weight intraperitoneally once a week. In order to achieve complete blinding, 10 ml/kg body weight of 0.9% NaCl was administered on the other eight injection days.

All substances were purchased at once and from the same vendors to ensure using the same LOTs and charge numbers to rule out charge effects.

**Tumor xenograft model**

In the PINPRICS-PS study BALB-C mice with a breast cancer xenograft consisting of commercially available 4T1 breast cancer cells were used. 4T1 cells (ATCC, Washington DC) are low immunogenic but highly malignant human breast cancer cells. Cells were injected at a dose of 5x 10^4^ cells into the subcutaneous fatty tissue of the lateral mammary gland of female BALB/c mice as described previously [[11](#_ENREF_11)]. The primary tumor was regularly measured with a caliper and treatment with chemotherapy and neuroprotective substances started seven days after tumor cell injection. To ensure that the primary endpoint on day 42 can be measured in the Kolliphor EL : Ethanol group, only 2.5x 10^4^ 4T1 cells were injected into animals from this group. The blinding of the groups was maintained by having these injections carried out by persons not otherwise involved in the experiment.

**Cell culture experiments**

Culture of 4T1 cells:

# **4T1 cells were obtained from American Type Culture Collection (ATCC, Manassas, VA) and cultivated as recommended by the manufacturer in RPMI-1640 medium (Merck / Sigma-Aldrich, Germany) supplemented with 10% fetal bovine serum and 1% peniclline/streptomycin (both BiochChrom, Germany). Cultures were kept at 37°C in a humidified atmosphere with 5% CO_2_.**

Cell viability assays:

Cytotoxicity of paclitaxel in cultured 4T1 cells was assessed with MTT and protease assays (Promega CytoTox-Fluor Assay, Germany) according to the manufacturers’ instructions and live/dead ratios of MTT/proteases assays were calculated and normalized to vehicle, as described previously [[12](#_ENREF_12)].

**Behavior analysis**

Prior to the experiment, animals were familiarized with the investigator by handling of animals for five days according to a previously specified handling protocol prior to the start of the experiment. During the experiments, experimenters randomly selected cages and animals in a laboratory with soundproof chambers.

RotaRod:

In PINPRICS-PS we assessed motor coordination using the rotarod performance test. Mice were placed on a rotating rod in individual compartments, with walls on both sides and in front of them (TSE Systems GmbH, Germany). Within 300 seconds the speed of the rotating rod increased from four rounds per minute (rpm) to a maximum speed of 40 rpm and the latency for the animal to fall off the rod was automatically recorded by a floor sensor. To allow mice to learn the task, animals were trained for four days with three trials per day, with a daily increase in the maximum time spent on the rod from 70 s per trial on day 1, to 140 s per trial on day 2, to 210 s per trial on day 3 and finally to 300 s per trial on day four. Mice that fell off the rod during training within the designated time were gently placed back on the rod. Mice were brought back to their home cage from the moving rod only to prevent animals from exhibiting dropping behavior. The baseline was recorded on the last day of training by measuring the initial latency to fall off the rod. Follow-up testing was done at 14, 28 and 42 days after initial PTX treatment to determine if motor coordination was affected. For each time point the results from three trials were averaged.

Von Frey Hair test:

In PINPRICS-PS mechanical allodynia was assessed using von Frey hairs and the up-down method, as described previously [[13](#_ENREF_13)]. The 50 % probability withdrawal threshold was determined. The mice were placed under an inverted plastic cage with a wire-mesh floor. Investigators underwent extensive training to apply the filaments to the center of the hind paws, gradually increasing pressure. Poking either hind paw evoked a flexion reflex followed by a clear withdrawal response. The value of each filament that evoked a withdrawal response was noted and the next lower value was used for the next round of testing. Four time points were recorded (baseline, day 14,28, and 42).

**Nerve conduction studies**

In PINPRICS-DC and PINPRICS-PS nerve conduction velocity (NCV) and sensory nerve action potential amplitudes (SNAP) of the caudal nerve were recorded in isoflurane anesthesia (1.3% to 1.7% in 50% O_2_) with a 2-channel portable electromyography and nerve conduction system (Berlin: Neurosoft 3102evo, Schreiber & Tholen Medizintechnik, Germany; Cologne and Essen: Dantec Keypoint G3, Natus, Planegg, Germany). We used antidrome measurements with pre-specified stimulation and recording electrode positions as well as pre-specified stimulation parameters as outlined in Supplemental Figure 1. Fifty stimuli with supramaximal stimulation intensity and a frequency of 1 Hz were averaged to measure SNAP and SCV at four time points (baseline, day 14, 28 and 42). The main endpoint of PINPRICS-DC was change in tail-nerve sensory nerve action potential amplitude. We observed statistically significant differences between the different trial sites regarding the absolute baseline SNAP amplitudes, which was due to differences in electrophysiological measurement apparatuses used. In order to enable comparisons between the different trial sites, an individual center specific mean baseline value was calculated and used to normalize data recorded at the consecutive time points.

**Sample size calculation and statistical analysis plan**

Detailed justification sample size PINPRICS-DC:

*Detailed Justification*

As we have three doses to select, the probability $p$ to select a correct dose of a given a medication regimen should be chosen such that

$$p^{3}=0.90\Longleftrightarrow p=\exp\left( \frac{\log\left( 0.9 \right)}{3} \right)\approx0.9654894.$$

For a given medication regimen assume

| $X_{1},\ldots, X_{n}, Y_{1},\ldots, Y_{n} \sim N\left( \mu,\sigma^{2} \right),$ | (suboptimal doses) |
| --- | --- |
| $Z_{1},\ldots, Z_{n} \sim N\left( \mu+\delta,\sigma^{2} \right), \delta>0,$ | (optimal dose) |

where all random variables are independent.

Then, for a fixed sample size $n$ the differences of sample means have the following distribution

$$\binom{\bar{D}_{n}^{ZX}}{\bar{D}_{n}^{ZY}}:=\binom{\bar{Z}_{n}-\bar{X}_{n}}{\bar{Z}_{n}-\bar{Y}_{n}} \sim N\left( \binom{\delta}{\delta} , \frac{\sigma^{2}}{n}\left( \begin{matrix} 2 & 1 \\ 1 & 2 \end{matrix} \right) \right).$$

This in turn implies

$$\mathbb{P}\left( \bar{Z}_{n}>\bar{X}_{n},\bar{Z}_{n}>\bar{Y}_{n} \right)\mathbb{=P}\left( \bar{D}_{n}^{ZX}>0,\bar{D}_{n}^{ZY}>0 \right).$$

If we choose$\delta=4$ and $\sigma=4$, the following R script

**library**(mvtnorm)

delta <- 4

sigma <- 4

Tau <- sigma^2*matrix(c(2,1,1,2),ncol=2,nrow=2)

max <- 15

prob <- rep(NA,max)

**for** (i **in** 1:max) {

prob[i] <- pmvnorm(lower=c(0,0),upper=c(Inf,Inf),mean=c(delta,delta),sigma=Tau/i)[1]

}

data.frame(n=1:max, prob)

readily provides a sample size of 9.

## n prob

## 1 0.6337020

## 2 0.7452036

## 3 0.8165829

## 4 0.8657672

## 5 0.9007794

## 6 0.9261635

## 7 0.9447835

## 8 0.9585527

**## 9 0.9687955**

## 10 0.9764497

Detailed justification sample size PINPRICS-PS:

*Detailed Justification*

Assume that the endpoint follows a normal distribution. We employed the power calculation approach as set forth in [1]. The corresponding R Code is given here.

**library**(mvtnorm)

set.seed(333)

power.mct <- **function**(mu,sigma,contrast,n,groups,alpha){

nn <- rep(n,groups)

V <- diag(1/nn)

df <- (groups-1)*n

cv <- C%*%V%*%t(C)

cr <- cov2cor(cv)

d <- C%*%mu

delta <- as.vector(d/ (sigma*sqrt(c(diag(C%*%V%*%t(C))))))

talpha <- qmvt(1-alpha, df=df, delta=rep(0,6), corr=cr, abseps=1e-09, maxpts=1e+05,tail="both.tails", type="Kshirsagar")$quantile

alpha <- 1-pmvt(lower=rep(-talpha,6),upper=rep(talpha,6),delta=rep(0,6),df=df,corr=cr,abseps=1e-09, maxpts=1e+06,type="Kshirsagar") # alpha

power <- 1-pmvt(lower=rep(-talpha,6),upper=rep(talpha,6),delta=delta,df=df,corr=cr,abseps=1e-09, maxpts=1e+06,type="Kshirsagar") # power

###

NOTE <- "n is number in *each* group"

METHOD <- "multiple contrast test power calculation"

structure(list(n = n, groups = groups, d=d, sigma = sigma, ncp=delta, talpha=talpha, alpha = alpha, power = power, note = NOTE,

method = METHOD), class = "power.htest")

}

C1 <- cbind(diag(rep(1,3)),rep(-1,3),rep(0,3))

C2 <- cbind(diag(rep(1,3)),rep(0,3),rep(-1,3))

C <- rbind(C1,C2)

power.mct(mu=c(30,30,30,26,26),sigma=4,contrast=C,n=14,groups=5,alpha=0.05)

power.mct(mu=c(30,30,30,26,26),sigma=4,contrast=C,n=15,groups=5,alpha=0.05)

The results are given here.

> power.mct(mu=c(30,30,30,26,26),sigma=4,contrast=C,n=15,groups=5,alpha=0.05)

multiple contrast test power calculation

n = 14

groups = 5

d = 4, 4, 4, 4, 4, 4

sigma = 4

ncp = 2.645751, 2.645751, 2.645751, 2.645751, 2.645751, 2.645751

talpha = 2.66802

alpha = 0.0499527

power = 0.885582

NOTE: n is number in *each* group

> power.mct(mu=c(30,30,30,26,26),sigma=4,contrast=C,n=15,groups=5,alpha=0.05)

multiple contrast test power calculation

n = 15

groups = 5

d = 4, 4, 4, 4, 4, 4

sigma = 4

ncp = 2.738613, 2.738613, 2.738613, 2.738613, 2.738613, 2.738613

talpha = 2.662242

alpha = 0.04998249

power = 0.9084805

NOTE: n is number in *each* group

Electronic trial database

Study data were collected and managed using REDCap electronic data capture tools hosted at a server of Charité Universitaetsmedizin Berlin [[16](#_ENREF_16), [17](#_ENREF_17)]. REDCap (Research Electronic Data Capture) is a secure, web-based software platform designed to support data capture for research studies, providing 1) an intuitive interface for validated data capture; 2) audit trails for tracking data manipulation and export procedures; 3) automated export procedures for seamless data downloads to common statistical packages; and 4) procedures for data integration and interoperability with external sources. Pseudonymized data were entered into REDCap by all study centers. Data export, cleaning and plausibility checks as well as data analysis was done by an independent unblinded biostatistician at Charité Universitaetsmedizin Berlin.

Statistical analysis and data presentation

Data is expressed as mean ± standard deviation respectively median with range and the manuscript was written in accordance with ARRIVE guidelines [[18](#_ENREF_18)]. Data analysis was completed before the researcher was unblinded. Due to the rapid loss of animals in the PINPRICS-PS and corresponding reduced group size, we decided according to the biostatistician´s advice to analyze efficacy endpoints at day 14 instead –as pre-specified- of day 42. Statistical analysis of the differences between treated versus control groups was performed as pre-specified with a multiple contrast test using a linear regression model adjusted for the baseline SNAP amplitude and center by means of a covariate (ANCOVA) with the multcomp package in R. *p* < 0.05 was considered statistically significant.

References:

1. Bretz F, Genz A, A. Hothorn L. On the Numerical Availability of Multiple Comparison Procedures. Biometrical Journal. 2001;43(5):645-56. doi: 10.1002/1521-4036(200109)43:5<645::aid-bimj645>3.0.co;2-f.
